# Epithelial-Mesenchymal Transition Drives Three-Dimensional Morphogenesis in Mammalian Early Development

**DOI:** 10.3389/fcell.2021.639244

**Published:** 2021-02-11

**Authors:** Galym Ismagulov, Sofiane Hamidi, Guojun Sheng

**Affiliations:** International Research Center for Medical Sciences (IRCMS), Kumamoto University, Kumamoto, Japan

**Keywords:** EMT, epiblast, pluripotency, polarity, gastrulation

## Abstract

From fertilization to onset of gastrulation, a mammalian embryo goes through several rounds of cellular morphogenesis resembling phenomena of epithelial-mesenchymal transition (EMT) and mesenchymal-epithelial transition (MET), collectively referred to as EMTs. How these EMT events play a role in shaping the three-dimensional (3-D) architecture of the developing embryo is not well-understood. In this review, we present a model in which cellular morphogenesis, represented primarily by dynamic changes in its epithelialization status, is the driving force of embryonic 3-D organization. This is achieved through the integration of three key components of mammalian early development, the pluripotency regulation, morphogenetic signaling, and biomechanical force anisotropy. Although cells in an early embryo do not exhibit full mesenchymal characteristics, our model underscores the importance of investigating molecular regulation of epithelial cell polarity and partial EMT/MET in understanding mammalian early development.

## Introduction

After fertilization, a mammalian embryo undergoes several rounds of early cleavages before acquisition of apicobasal polarity through compaction and segregation of inner and outer cells through asymmetric divisions (Humiecka et al., 2017). Cells inheriting the apical cortical domain at 8-cell to 16-cell division will reestablish polarity and position themselves in the outer layer of the embryo and will be biased to become trophectoderm (TE) progenitors. The rest of the blastomeres lack apical surface and apicobasal polarity and will be positioned in the inner part of the embryo and eventually contribute to the inner cell mass (ICM) (Maître et al., 2016). The ICM cells will then sort out, in an actin-dependent manner, to give rise to an epithelialized primitive endoderm (hypoblast) (Bedzhov and Zernicka-Goetz, 2014), whereas the remaining epiblast precursors maintain their non-epithelial characteristics. Those epiblast precursors will then undergo an MET process to form an apicobasally polarized epiblast epithelium and generate a pro-amniotic cavity as a consequence of this process. The epithelialized epiblast subsequently goes through an EMT process during gastrulation, generates the mesoderm and definitive endoderm, and lays down the foundation of the three-germ layer and three-dimensional (3-D) mammalian body plan.

Up till the onset of gastrulation, the architecture of a mammalian embryo largely reflects morphogenetic features of its constituent cells, whereas more complex interactions involving cells, tissues, and tissue compartments govern gastrulation and post-gastrulation morphogenesis. In this review, we aim to synthesize published data on early mammalian development with respect to the regulation of cellular morphogenesis and an embryo's 3-D organization, with an emphasis on the interplay between epithelial (E) and/or mesenchymal (M) status, pluripotency, biomechanical forces and morphogen signaling.

## Inter-Dependency Between Cellular Morphology and Pluripotency

**Figure 1:**
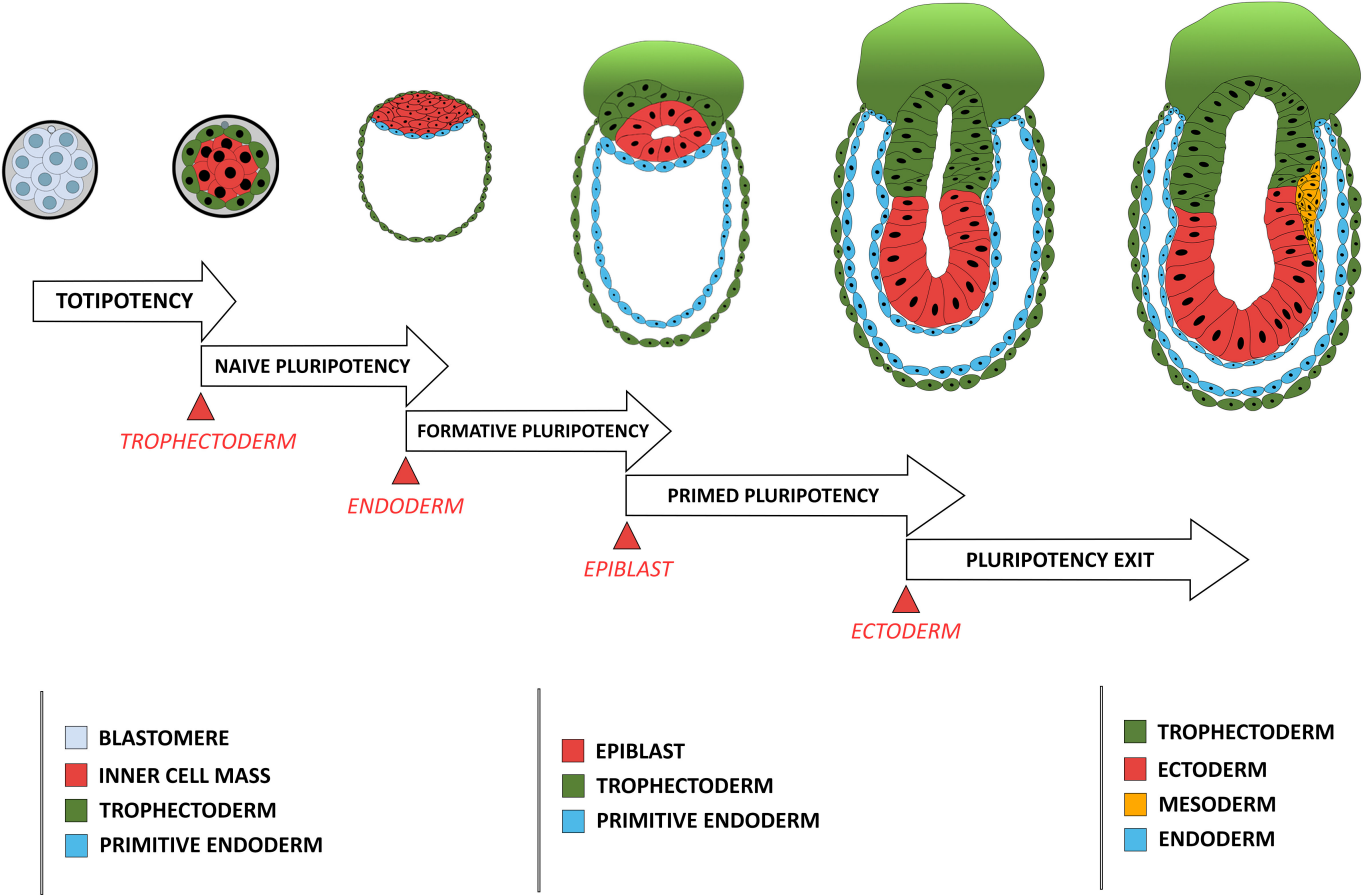
Comparison between the developmental stage of a mouse embryo and the concomitant restriction in cellular potency. Each restriction in cellular potency is associated with the creation of an embryonic structure through an epithelialization process: the totipotency is lost when the trophectoderm epithelializes; the naïve pluripotency loss corresponds to the epithelialization of the endoderm; the formative state loss is concomitant with the epithelialization of the epiblast; and the primed pluripotency loss corresponds to the acquisition of a fully epithelial organization by the epiblast leading to pluripotency exit, a stage poised for differentiation including ectoderm-fated epithelium formation and mesendoderm induction through another round of EMT.

Cellular potency is defined by its ability to differentiate into various cell types either *in vivo* or *in vitro*, varying from totipotency, corresponding to the status of blastomeres before lineage segregation between the TE and ICM, to unipotency which corresponds to cells that can differentiate only into a single cell type (Figure 1). Status of a cell's potency in early development reflects both the embryo's developmental stage and the cell's morphogenetic status. For instance, loss of totipotency of early blastomeres is associated with segregation of TE and ICM cells, and the restriction of potency is more pronounced for the outer, apicobasally polarized cells (TE) than the non-polarized ICM cells, which remain pluripotent and can give rise to all cell lineages in an adult body.

Pluripotency was conceptualized based on differentiation capacity (either *in vitro* or *in vivo*) of cells isolated *in vivo* and maintained *in vitro*. Recent efforts have been made to associate pluripotency with features of either the original developmental stage during pluripotent cell derivation or the putative developmental stage after those pluripotent cells have achieved a steady state under a given culture condition. Four pluripotency states have so far been characterized using cell morphological and molecular criteria. The first one is termed “naïve pluripotency,” which corresponds to the ICM cell after TE specification and segregation. Naive pluripotent cells do not have apicobasal polarity. They express a particular set of associated genes (e.g., REX1) (Ghimire et al., 2018). The second type is termed “formative pluripotency,” which corresponds to epithelializing epiblast precursors at the time of primitive endoderm specification and segregation. The formative pluripotent cells retain germline differentiation capability and are defined molecularly by OTX2 upregulation (Stumpf and MacArthur, 2019; Kinoshita et al., 2020). The third type is “primed pluripotency,” corresponding to a partially epithelialized epiblast prior to gastrulation. These cells have many epithelial characteristics (although they are not fully epithelialized) and have lost the capacity to contribute to the germline or to make chimeras (a phenomenon likely related to their epithelial status). Primed pluripotent cells have a differentiation bias toward specific cell linages that reflects their developmental progression along the antero-posterior axis of the embryo (Shahbazi et al., 2017). Finally, the fourth stage is the “pluripotency exit.” Cells at this stage are on the verge of differentiation (Thakurela et al., 2019) and are characterized by a progressive reduction of the core pluripotency factors such as OCT4, NANOG and SOX2. These cells can still reverse their pluripotent status upon changes in culture conditions (Hamidi et al., 2020) or developmental cues (Tian et al., 2019).

In human development, these transitions are also supported by progressive epithelialization of the epiblast and its segregation into the embryonic epiblast (giving rise to future three-germ layers) and the amniotic epiblast (contributing to the ectoderm portion of the amnion), and the appearance of the proamniotic cavity. The molecular mechanisms that link changes in cellular morphology and cellular potency are still not well-explored. GRHL2, an epithelial state inducer, has been reported to control the expression of a subset of the pluripotent network genes upon transition from naive to primed pluripotency state (Chen et al., 2018). Conversely, MCRS1, a mesenchymal state inducer (Liu et al., 2014), is required to maintain the epiblast lineage after trophectoderm and primitive endoderm specification, suggesting that the ability to retain an non-polar mesenchymal-like phenotype in the ICM is critical (Cui et al., 2020).

## Cell Morphology and Biomechanical Forces

Biomechanical forces in the embryo are a function of two main cellular features: cell motility and cell deformation. They play critical roles in shaping the embryo's 3-D architecture prior to gastrulation. During the process of TE and ICM segregation (Marikawa and Alarcón, 2009), mechanical forces are directly responsible for their position and fate specification through regulation of the orientation of asymmetric divisions. Cells located on the embryo's surface retain contractile abilities and differentiate into TE cells, whereas cells on the center, without contractile abilities, become the ICM (Bissiere et al., 2018). Consecutively, during blastocyst cavity formation, trophectoderm epithelial integrity associated with directional fluid transport results in the establishment of a blastocyst luminal pressure that drives further lumenogenesis and embryo growth (Wang et al., 2018; Chan et al., 2019).

**Figure 2:**
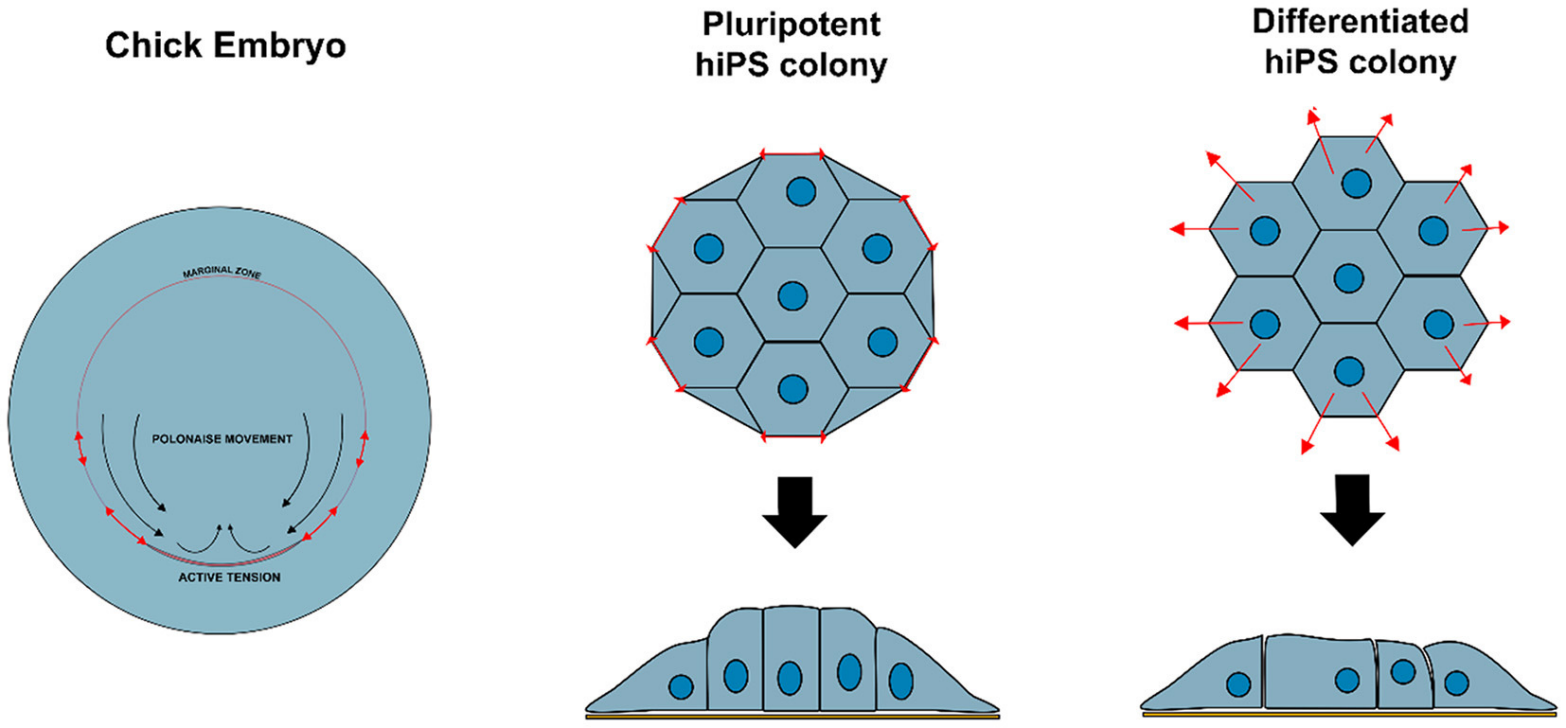
Variation in biomechanical stress can modulate cell behavior and identity. Contractile actomyosin ring in the marginal zone of a developing chicken embryo induces cellular deformations that affect epiblast structure and promote coordinated circular cell migration and initiation of primitive streak. Similar structure is observed at the edge of hiPS cell colonies *in vitro*, resulting in tensile forces that maintain specific cellular organization and enforce pluripotency of the hiPSC colony.

Non-polarized naïve epiblast precursors at this stage do not respond to mechanical stimuli (Verstreken et al., 2019). Later on, at the peri-implantation stage, upon ECM-mediated epiblast epithelialization, the luminal pressure will induce apical surface repulsion, which contributes to the fusion of multiple rosette-like clusters and to the establishment of inverted cup shaped-morphology of the mouse embryo (Christodoulou et al., 2018; Dokmegang et al., 2020). In parallel, the progression of epiblast cells from non-polarized naïve pluripotent cells to apicobasally polarized primed cells enables biomechanical signaling to play a role in epiblast cells' fate decisions (Verstreken et al., 2019). An amniote embryo's shape at the peri-gastrulation stage exhibit species-specific variation (Sheng, 2015). As a consequence, the underlying mechanical forces and their interplay with cellular morphogenesis can also vary. In the chick embryo, for example, where the peri-gastrulation embryo's shape can be likened to a disc, a supracellular actomyosin ring located in the marginal zone, at the edge of the embryonic tissue, controls the embryo global geometry. This ring also induces cellular deformations that are transmitted locally though the epiblast epithelial structure, inducing “polonaise-like” coordinated cellular migration and initiation of the primitive streak (Saadaoui et al., 2020). The “global” architecture of the pre-gastrulating chick embryo is somewhat similar to the “embryonic disc” seen in some mammalian groups, including in the human, and interestingly, a similar supracellular actin structure has also been reported to exist at the edge of disc-shaped human pluripotent stem cell (PSC) colonies *in vitro* (Närvä et al., 2017) (Figure 2). However, its existence *in vivo* (in the human embryo) or its causal relationship with human primitive streak initiation is unclear.

*In vitro* findings suggest that human PSCs can adopt a spectrum of morphological identities between fully epithelial and partially mesenchymal *in vitro*, as a consequence of differences in ECM compositions and concentrations (Hamidi et al., 2020), possibly reflecting ECM-dependent localized variations in migratory potential and epithelialization status of epiblast cells *in vivo* (Nakaya et al., 2013). It has been reported that basement membrane composition can influence PSC's epithelialization (Futaki et al., 2019; Hamidi et al., 2020) or induce EMT upon basement membrane breakdown (Nakaya et al., 2008), consistent with the fact that ECM's heterogeneity increases as embryonic development proceeds (Loganathan et al., 2016; Futaki et al., 2019). Spatiotemporal-specific ECM composition can be the blueprint of biomechanical force anisotropy and of an embryo's 3-D structure by providing a highly-specific local environment adequate for generating biomechanics-mediated signaling complexity (Loganathan et al., 2014).

## Cell Morphology and Response To Morphogens

**Figure 3:**
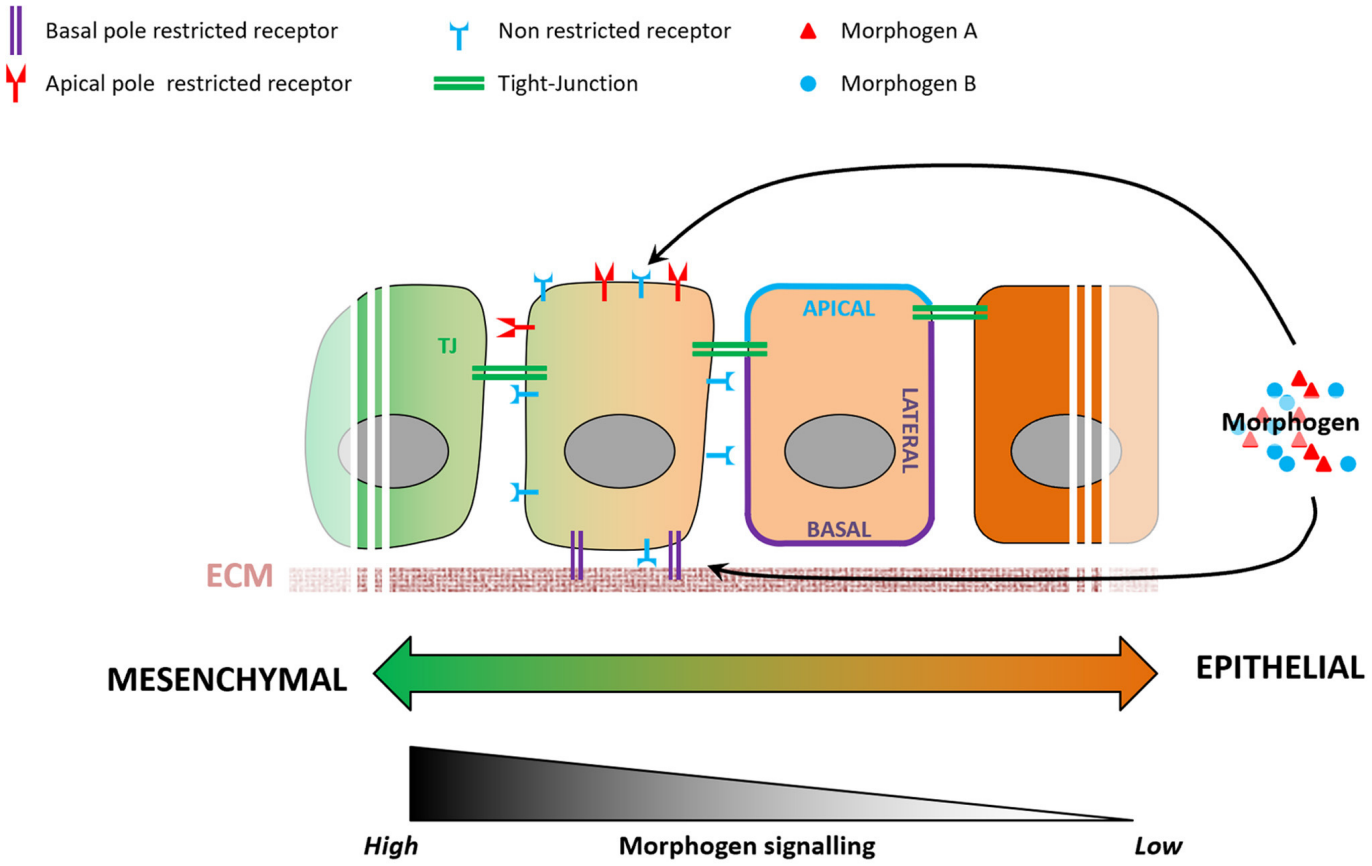
Variations in epithelial organization can modulate levels of cellular response to a given morphogen. During the epithelialization process, tight junctions act as impermeable seals that compartmentalize the apical and basolateral membranes of a cell. As a consequence, a morphogen present at the apical pole will not be able to reach receptors localized on the basolateral membrane, and vice-versa. Some receptors are known to be localized specifically to either the apical or basolateral plasma membranes, and variations in cell's epithelial organization will result in different levels of signaling pathway activation.

Morphogen signaling regulates many aspects of embryonic development (Tuazon and Mullins, 2015), action of which remains poorly understood. For instance, cells exposed to the same signals exhibit distinct fates depending on their spatiotemporal information in the embryo (Morgani and Hadjantonakis, 2020), and cellular geometry controls the level of response to morphogens in the mouse embryo (Zhang et al., 2019). Epithelialization-associated specification of apical and basolateral compartments of a cell involves the establishment of apical junctions as barriers to molecular diffusion. Morphogen availability and morphogen receptors are known to localize preferentially to either the apical or the basolateral membranes of the epithelium (Yin et al., 2017), resulting in a likewise compartmentalized molecular and subcellular response to a given morphogen (Figure 3). BMP receptors, for example, are present in mesodermal cells at levels similar to those in epiblast cells, but the BMP signaling activity is higher in the mesoderm cells (Morgani et al., 2018). Transition between epithelial and mesenchymal states of the same cell population (e.g., EMT during gastrulation) involves loss of apicobasal polarization, which may lead to more BMP receptors being exposed to BMP morphogen in mesoderm cells and enhance cellular response to it. *In vitro* models of human epiblast formation and differentiation allow us to investigate this question in more detail (Warmflash et al., 2014; Williams and Solnica-Krezel, 2017; Hamidi et al., 2020). In hESC colonies, differences in receptor accessibility due to apicobasal polarization led to the establishment of a gradient of BMP signaling activation, resulting in differential commitment of PSCs to the three germ layers (Etoc et al., 2016; Hamidi et al., 2020).

Additional levels of complexity in modulating differential effect of morphogen on cellular fate specification can be achieved by ECM heterogeneity and its role in mediating morphogen availability or receptor activation at the basolateral side. In addition to controlling cells' epithelial or mesenchymal status, the ECM can also act as a filtering mesh that can slow down, concentrate or biochemically modulate the action of a given morphogen. As a consequence, cells with similar morphological characteristics and morphogen exposure can display different responses depending on their ECM composition.

## What Can We Learn From This EMT/MET-Centric Model?

**Figure 4:**
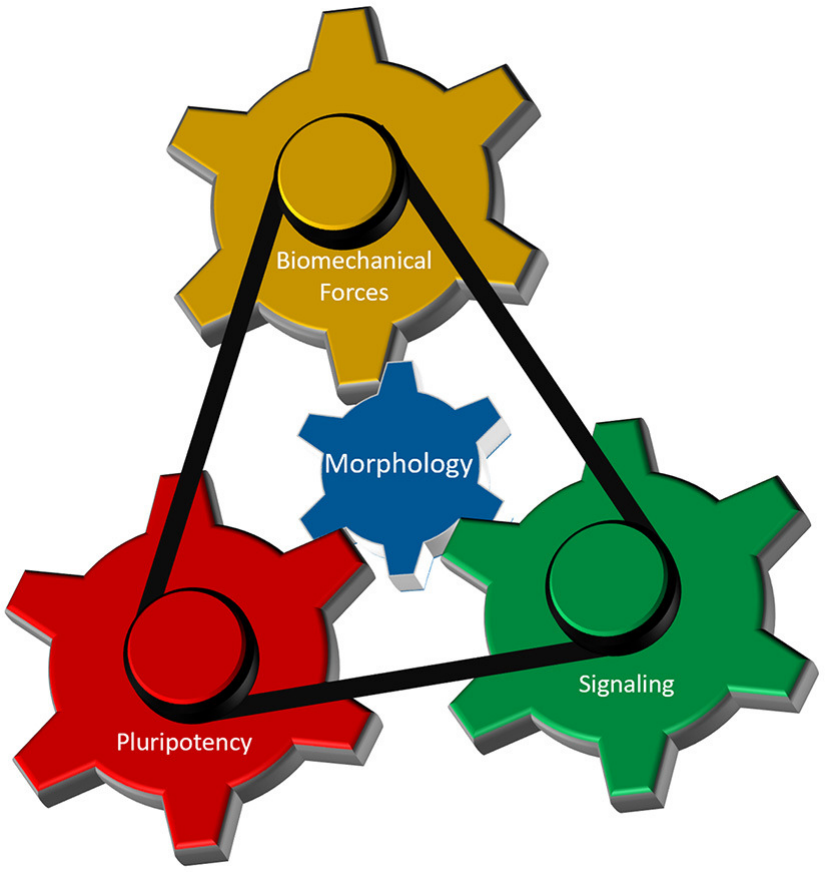
Cellular morphology as the fundamental parameter that coordinates different regulatory components of 3-D morphogenesis. Biomechanical forces, cellular potency and molecular signaling are the main components that dictate early morphogenesis of an embryo. Cellular morphology, defined by its epithelial and/or mesenchymal status, interacts with each of those three components to induce local variations in cellular responses necessary for the formation of highly reproducible structure in an early embryo.

As discussed above, interplay between cellular morphology, pluripotency, biomechanical forces, and morphogen signaling is critical for establishing proper 3-D architecture of a developing embryo (Figure 4). However, the mechanistic relationship between these events remains elusive. Why do cells require concurrent morphological transitions in order to achieve pluripotency changes? Do mechanical forces affect morphogen signaling and pluripotency levels, or is it the signaling that results in a specific manifestation of mechanical forces that in turn regulate cellular behavior? According to a recent model (Das et al., 2019), biomechanical forces can be a secondary consequence of short-range morphogen signaling that ensures the robustness and long-range transmission of information necessary for proper embryo-level morphogenesis. To accomplish this, cells are required to have appropriate cell-cell and cell-ECM interactions, permitting transmission of the mechanical signals. Thus, cellular morphology may act as a signal integrator that can receive, process, and send specific information among cells located in neighboring tissues.

Indeed, large-scale morphogenetic movements (e.g., during primitive streak formation) involve oriented cellular intercalation and morphology changes that can be understood from the perspective of polarized cytoskeletal organization and directional biomechanical tension (Ferro et al., 2020), associated with the supracellular organization of the actin-myosin cytoskeletal network of the epiblast epithelium (Hamidi et al., 2019; Ferro et al., 2020). However, in view of the robustness and speed of signal transduction through mechanical means, it is still unclear how cells can sense the requirement for morphological changes and effectively communicate with each other to construct elaborate, supracellular structures. A possible hypothesis, from analysis of zebrafish gastrulation, could be that there is heterogeneity in such integration and that some cells respond and relay more readily a given signal to their neighbors (Das et al., 2019).

**Figure 5:**
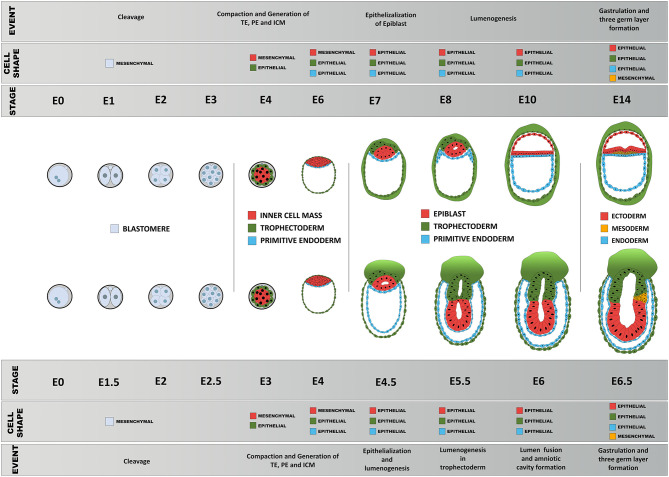
Schematic diagram of comparison of early morphogenesis in the human and mouse. Upon fertilization, mouse and human embryos undergo a series of morphological changes, primarily as a consequence of the interplay between their epithelial and mesenchymal status, pluripotency, biomechanical forces, and morphogen signaling. Blastomeres show little apicobasal polarization before compaction between 8 → 16 cell stage that results in the first lineage specification: epithelialized trophoblast and non-polar inner cell mass. The primitive endoderm precursors then undergo epithelialization followed by a gradual polarization process of the epiblast precursors, resulting in formation of the proamniotic cavity and an epithelial epiblast that will be maintained until the next major transformation event during gastrulation.

During early embryonic development, it is unclear why cells need to undergo successive morphological transformation in order to achieve the complexity in cellular differentiation. Is it a necessity to ensure proper control over the morphogenetic process or just a direct consequence of morphology changes? The former would imply that it is necessary to restrict cellular potency as embryonic morphogenesis progresses in order to reduce potential differentiation errors. However, this paradigm seems to be contradicted by a recent study on totipotency loss and trophectoderm differentiation in which the authors showed that ICM-derived naïve PSCs retain the ability to differentiate into the trophectoderm lineage (Guo et al., 2020). The latter implies that acquisition of epithelial features is sufficient to trigger differentiation process(es) that would restrict their potency. This scenario may be a more realistic one as a recent study showed that attachment of the cellular plasma membrane to the submembrane cortex protects naïve PSCs from progressing to a primed state or further (Bergert et al., 2020). Using Ezrin mutant proteins to modulate the membrane-to-cortex attachment (MCA), the authors showed that a high MCA inhibited PSC differentiation, whereas a low MCA resulted in a permissive state that allows differentiation. Moreover, strong membrane-to-cortex attachment is known to maintain cellular polarity and protect cells from morphologic transition (Houk et al., 2012; Schneider et al., 2013). This suggests that in the case of MCA, cells are preferentially using physical forces as checkpoints to control their progression through important morphogenetic events (Figure 5). This and other similar hypotheses could be tested in the future by combining *in vitro* models of early epiblast-like PSC organization (Deglincerti et al., 2016) with the use of biomaterials capable of dynamic modulation of their physical properties (LeValley and Kloxin, 2019). Such approaches would enable a powerful *in vitro* platform, with high reproducibility and programmability, to investigate how mechanical signals are integrated into the biochemical network that regulates cellular specification and tissue patterning and whether cell morphological changes might be the missing link reconciling how mechanical and chemical signals influence cell identity during development.

## Data Availability Statement

The original contributions presented in the study are included in the article/supplementary material, further inquiries can be directed to the corresponding author/s.

## Author Contributions

GS conceived the manuscript topic. GI, SH, and GS discussed about the writing and wrote the manuscript. All authors agreed on the content of manuscript.

## Conflict of Interest

The authors declare that the research was conducted in the absence of any commercial or financial relationships that could be construed as a potential conflict of interest.
